# A minimum core outcome set for clinical trials on non-minimal-invasive off- or on-pump cardiothoracic surgery

**DOI:** 10.1186/1745-6215-16-S3-O1

**Published:** 2015-11-24

**Authors:** Carina Benstöm, Ajay Moza, Rüdiger Autschbach, Christian Stoppe, Andreas Goetzenich

**Affiliations:** 1 Department of Thoracic and Cardiovascular Surgery, University Hospital RWTH Aachen, Aachen, Germany; 2 Department of Anaesthesiology, University Hospital RWTH Aachen, Aachen, Germany

## Background

Cardiovascular disease (CVD) is a major contributor to the burden of disease and the number one cause of death worldwide. From 1990 until today, more people have died from coronary heart disease than from any other cause. CVD is regularly treated with minimal or non-minimal-invasive off- or on-pump cardiothoracic surgery and interventions related to the outcome of the surgical procedures are regularly evaluated in clinical trials, but heterogeneity in outcome reporting hinders comparison of interventions and limits the ability of research synthesis. This problem is encountered by core outcome sets (COS) that should be measured and reported – as a minimum – in all clinical trials for a specific clinical field.

## Method

We are developing a COS for clinical trials measuring the efficacy and effectiveness of pre-, intra- or postsurgical interventions in non-minimal-invasive off- or on-pump cardiothoracic surgery (elective and emergency procedures, excluding transplants, participants > 18 years). Recommendations on COS development given by the Core Outcome Measures in Effectiveness Trials (COMET) Initiative and the Outcome Measures in Rheumatology (OMERACT) Initiative were followed. We will reach consensus on core domains in a 3-round eDelphi exercise involving adult patients in need or after cardiothoracic surgery, cardio-thoracic surgeons, anaesthesiologists, nursing staff and researchers with expertise in this field of research. We aim to agree on measurement instruments for each of the identified core domains subsequently.

## Results

The eDelphi is currently being carried out. We will present identified core outcome domains at the COMET V Meeting.

## Conclusion

The proposed COS aims to provide methodological guidance in future cardiothoracic surgical clinical trials. This does not imply that primary outcomes should always and exclusively be those of the COS. However, to assure the comparability of results across trials the outcomes included in this COS should be considered for inclusion besides measuring trial-specific clinical endpoints.

